# CHIP E3 ligase mediates proteasomal degradation of the proliferation regulatory protein ALDH1L1 during the transition of NIH3T3 fibroblasts from G_0_/G_1_ to S-phase

**DOI:** 10.1371/journal.pone.0199699

**Published:** 2018-07-06

**Authors:** Qasim A. Khan, Peter Pediaditakis, Yuryi Malakhau, Amin Esmaeilniakooshkghazi, Zahra Ashkavand, Valentin Sereda, Natalia I. Krupenko, Sergey A. Krupenko

**Affiliations:** 1 Nutrition Research Institute, University of North Carolina at Chapel Hill, Kannapolis, North Carolina, United States of America; 2 Department of Nutrition, University of North Carolina at Chapel Hill, Chapel Hill, North Carolina, United States of America; University of Pittsburgh, UNITED STATES

## Abstract

ALDH1L1 is a folate-metabolizing enzyme abundant in liver and several other tissues. In human cancers and cell lines derived from malignant tumors, the *ALDH1L1* gene is commonly silenced through the promoter methylation. It was suggested that ALDH1L1 limits proliferation capacity of the cell and thus functions as putative tumor suppressor. In contrast to cancer cells, mouse cell lines NIH3T3 and AML12 do express the ALDH1L1 protein. In the present study, we show that the levels of ALDH1L1 in these cell lines fluctuate throughout the cell cycle. During S-phase, ALDH1L1 is markedly down regulated at the protein level. As the cell cultures become confluent and cells experience increased contact inhibition, ALDH1L1 accumulates in the cells. In agreement with this finding, NIH3T3 cells arrested in G_1_/S-phase by a thymidine block completely lose the ALDH1L1 protein. Treatment with the proteasome inhibitor MG-132 prevents such loss in proliferating NIH3T3 cells, suggesting the proteasomal degradation of the ALDH1L1 protein. The co-localization of ALDH1L1 with proteasomes, demonstrated by confocal microscopy, supports this mechanism. We further show that ALDH1L1 interacts with the chaperone-dependent E3 ligase CHIP, which plays a key role in the ALDH1L1 ubiquitination and degradation. In NIH3T3 cells, silencing of CHIP by siRNA halts, while transient expression of CHIP promotes, the ALDH1L1 loss. The downregulation of ALDH1L1 is associated with the accumulation of the ALDH1L1 substrate 10-formyltetrahydrofolate, which is required for *de novo* purine biosynthesis, a key pathway activated in S-phase. Overall, our data indicate that CHIP-mediated proteasomal degradation of ALDH1L1 facilitates cellular proliferation.

## Introduction

**Fig 1:**
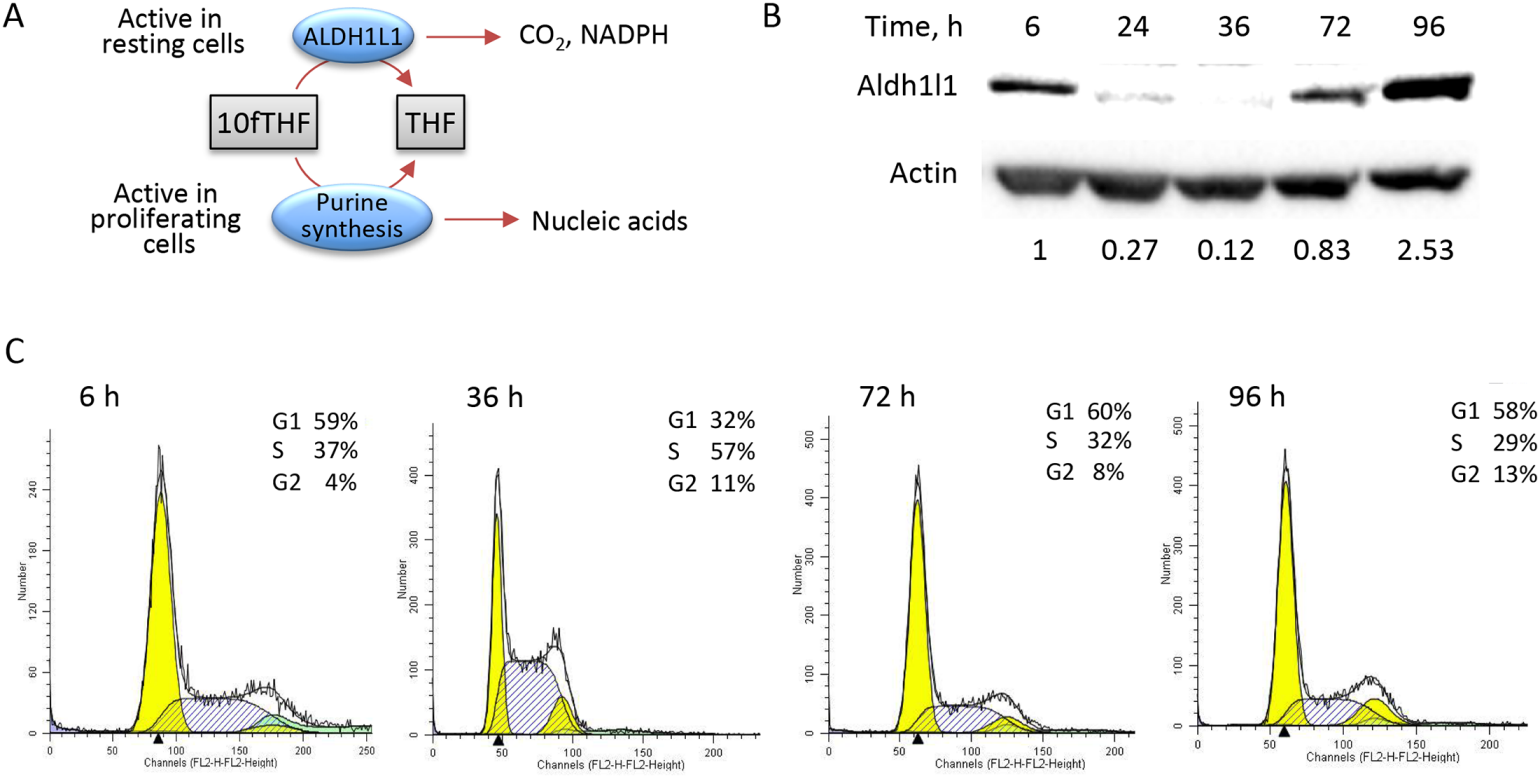
Levels of ALDH1L1 protein fluctuate in proliferating NIH3T3 cells. **A**, schematic depicting the ALDH1L1 metabolic pathway (the enzyme converts 10-formyl-THF to THF and CO_2_ simultaneously producing NADPH; this pathway competes with *de novo* purine biosynthesis for the same substrate, 10-formyl-THF). **B-C**, levels of ALDH1L1 in proliferating NIH3T3 cells during the cell cycle progression. Time points on graphs correspond to those on the blot and indicate hours after splitting confluent cell culture. Quantification of ALDH1L1 bands (arbitrary densitometry units) normalized to actin is shown. Cell cycle data were analyzed using ModFit software.

The folate metabolizing enzyme ALDH1L1 (10-formyltetrahydrofolate dehydrogenase) converts 10-formyltetrahydrofolate to tetrahydrofolate (THF) in a NADP^+^-dependent reaction (Fig 1A) [1]. This reaction controls the overall level of folate-bound one-carbon groups by removing them as CO_2_. Therefore, the ALDH1L1 reaction limits the flux of one-carbon groups through the folate-dependent biosynthetic pathways, which include *de novo* purine and thymidylate biosynthesis as well as the conversion of homocysteine to methionine [2, 3]. Also, ALDH1L1 acts to regenerate NADPH in the final step of the carbon oxidation [1]. Thus, the enzyme is viewed as a switch from a biosynthetic to an energy-producing pathway. Forced expression of the enzyme in ALDH1L1-deficient cultured cell lines depletes 10-formyl-THF and 5-methyl-THF pools, leads to a drop in purine nucleotides, and impairs homocysteine re-methylation [4–6]. This supports the role of ALDH1L1 in limiting the biosynthetic and proliferative capacity of the cell.

It has been also demonstrated that the expression of ALDH1L1 is ubiquitously lost in cancer cell lines [7]. Re-expression of the protein in ALDH1L1-deficient cancer cells inhibits proliferation and induces apoptosis, effects attributed to the decrease *de novo* purine biosynthesis [4, 7, 8]. The *ALDH1L1* gene is also under-expressed in human cancers compared to respective normal tissues, with certain malignant tumors completely lacking the ALDH1L1 protein [7, 9–14]. Silencing of this gene in cancers is achieved by the CpG island methylation in the promoter region [10–13]. It has been suggested that the silencing provides a selective advantage to cancer cells by removing biosynthetic limits on proliferation [7, 10]. Based on these findings, ALDH1L1 is considered as a putative tumor suppressor [7, 10, 12]. It is not clear whether the antiproliferative effect of ALDH1L1 is mostly limited to cancer cells or if the enzyme also participates in regulating the proliferation of normal cells. The findings that ALDH1L1 is highly expressed in regenerating liver, several transformed fibroblast cell lines and hemangioma, a benign tumor originated from endothelial cells, support the hypothesis that the ALDH1L1 antiproliferative effect is cancer specific [7, 10]. On the other hand, the cell-specific up-regulation of ALDH1L1 in PAX3-negative radial glia during central nervous system development correlates with reduced proliferation of cells at the midline of the neural tube during early murine embryogenesis [15]. Overall, these studies suggest that ALDH1L1 functions to suppress excessive proliferation in a folate-responsive manner during development.

Here we report that levels of ALDH1L1 protein in NIH3T3 cells oscillate during the cell cycle. ALDH1L1 is markedly decreased during active DNA biosynthesis (S-phase) and is increased as cells exit the cell cycle and become quiescent (G_0_-phase). The present study further demonstrates that the ubiquitin-dependent proteasomal degradation pathway participates in the rapid removal of ALDH1L1 protein in actively proliferating fibroblasts.

## Materials and methods

### Reagents

General purpose reagents were purchased from Sigma or ThermoFisher Scientific unless otherwise specified. Kits and reagents for ubiquitin-specific assays were obtained from Boston Biochem (Cambridge, MA).

### Cell culture

Cell media and reagents were purchased from Life Technologies (Carlsbad, CA, USA). Immortalized NIH3T3 mouse embryonic fibroblasts and AML12 cells were obtained from ATCC (Manassas, VA, USA). Cells were grown in DMEM, 1% of PSN antibiotic mixture, and 10% (v/v) calf bovine serum (Sigma) in a humidified atmosphere at 37 °C and 5% CO_2_.

### Immunoblot assays

NIH3T3 cells were lysed in RIPA buffer containing 1x protease inhibitor cocktail (Sigma). Cell lysates were subjected to SDS-PAGE (20 μg of the total protein loaded per lane) followed by Western blot assays with corresponding antibodies. ALDH1L1 was detected using in-house rabbit polyclonal antibody (1:10000) generated against purified recombinant rat ALDH1L1 [7, 16]. Primary antibodies used in this study are listed in S1 Table. All in-house antibodies have been verified in our previous studies [5, 17, 18]. HRP-conjugated secondary goat anti-rabbit IgG antibody and goat anti-mouse IgG antibody were from Abcam (both used at 1:7000). A Hybond TM-ECL nitrocellulose membrane (GE Healthcare Life Sciences, Pittsburg, PA, USA) and Pierce ECL detection kit (ThermoFisher Scientific, Waltham, MA, USA) were used.

### Flow cytometry

NIH3T3 cells were plated in 150 mm^2^ cell culture dishes at about 25% confluency. Cells were harvested 6–96 h later and processed for flow cytometry analysis. Briefly, harvested cells were washed with PBS, pH 7.4, and fixed in 70% ice-cold ethanol. Prior to analysis, cells were washed with PBS, treated with RNase A (3 U/ml) at 37 °C for 15 min, stained with PI (50 μg/ml in 100 mM Tris-HCl buffer pH 7.4 containing 150 mM NaCl and 0.1% Tween-20) for 20 min. The cell cycle arrest was achieved by: serum starvation (G_0_/G_1_ arrest, 1% serum for 20 h); double thymidine block (S-phase arrest); or nocodazole treatment after double thymidine block (G_2_/M arrest) [19]. For the double thymidine block, 40% confluent cells were treated with 2 mM thymidine for 14 h, washed once with PBS, and placed on standard media supplemented with 24 μM deoxycytidine for 9 h. Then 2 mM thymidine was added and cells were analyzed 14 h later. For the G_2_/M arrest, cells were released from double thymidine block by incubating for 2 h in standard media supplemented with 24 μM deoxycytidine, then 100 nM nocodazole was added, and cells were analyzed 10 h later. Flow cytometry analysis was performed on Becton Dickinson FacsAria III cell sorter, AMNIS flow cytometer (EMD Millipore), or CytoFLEX flow cytometer (Beckman Coulter). Data analysis was performed using ModFit (Becton Dickinson), FlowJo (FlowJo, LLC) or FCS Express (De Novo Software) software. About 10^6^ cells were used for analysis.

### Confocal microscopy

Cells were seeded in Lab-Tek II Chamber (Nalge Nunc International, Rochester, NY, USA), fixed with 3.7% methanol-free formaldehyde for 10 min, permeabilized with 0.1% Triton X-100 for 5 min and incubated with 3% BSA in PBS for 45 min. Slides were stained with ALDH1L1-specific in-house rabbit antibody (1: 1000) and PSMA6-specific (proteasome 20S alpha 6 subunits) mouse monoclonal antibody (OriGene, cat# TA800104; 1:100) at 4 °C overnight, washed three times with PBS and then incubated with secondary chicken anti-rabbit antibody conjugated with Alexa Fluor 488 (Thermo Fisher Scientific, cat. # A-21441; 1: 200) and goat anti-mouse IgG2a secondary antibody conjugated with Alexa Fluor 555 (Thermo Fisher Scientific, cat. # A21137; 1: 200) in dark chamber at room temperature for 1 h. Slides were washed twice with PBS and mounted with ProLong Gold Antifade Mountant with DAPI (Thermo Fisher Scientific). Images were captured using confocal laser scanning microscope Olympus FluoView FV10i (Olympus, Tokyo, Japan).

### Pull-down of ALDH1L1 interacting proteins using affinity column

Folate affinity resin was prepared using folinic acid as we previously described [20]. Human ALDH1L1 was expressed in insect cells using a baculovirus expression system and purified as we described [21]. Purified recombinant ALDH1L1 (about 4 mg) was loaded on the column (1.5 ml of the affinity resin). A549 cells (250 x 10^6^) were lysed in loading buffer (20 mM Tris-HCl buffer, pH 7.8, containing 150 mM NaCl, 10 mM 2-mercaptoethanol, 0.1% Triton X-100) containing 1% of protease inhibitor cocktail (Sigma), 1% of phosphatase inhibitor cocktail (Roche) and 10 μg/ml DNase-I. The sample was processed using Dounce homogenizer and sonicated. Insoluble components were removed by centrifugation (16,000xg for 20 min) and soluble lysate was loaded to the affinity column preloaded with ALDH1L1. The column was washed stepwise with 10 ml of loading buffer, 12.5 ml of the buffer containing 0.5 M KCl, and 7.5 ml of the buffer containing 2.0 M KCl. In the control experiment, pull-down was performed using the same affinity column in the absence of ALDH1L1. The 2.0 M KCl elution was concentrated to about 3.5 mg/ml protein and analyzed by LC/MS-MS.

### LC/MS-MS

LC/MS-MS experiments were carried out as we previously described [22] using services of the Proteomics Mass Spectrometry Core Facility, Medical University of South Carolina.

### Pull-down of ALDH1L1 from NIH3T3 cells using affinity column

The lysate of NIH3T3 cells was prepared as describe above for A549 cells. The lysate was loaded on the folate-affinity column (as in the control experiment described above). After washing the column with 10 ml of loading buffer, elution was carried out using a stepwise increased concentration of KCl (0.5, 1.0 and 2.0 M). Additional two steps included the elution with loading buffer/2.0 M KCl containing 5 and 20 mM folic acid. Eluted samples were concentrated to about 1 mg/ml protein using Millipore Amicon Ultra-4 Centrifugal Filter Units and analyzed by SDS-PAGE/Western blotting.

### Ubiquitination assay

*In vitro* ubiquitination was performed using CHIP Ubiquitin Ligase kit -Glow-Fold Substrate (Boston Biochem, Cambridge, MA) according to the manufacturer’s protocol. Briefly, in a 30 μl reaction mix, 3 μl of 10x reaction buffer, 3 μl of 10X Mg^2+^-ATP solution, 3 μl 10x HSP70/HSP40 mix, and the indicated amounts of recombinant ALDH1L1 were added, and the reaction mix was heated at 43 °C for 7 min, tubes were immediately transferred to ice for 10 min. Tubes were spun briefly, and the following reagents were added: 3 μl of 10x E1 enzyme, 3 μl of 10x E2 enzyme, and 3 μl of 10x CHIP. The ubiquitination reaction was initiated by adding 3 μl of 10x ubiquitin solution. The reaction tubes were incubated at 37 °C for 60 min. In a separate experiment, ATP, E1 or E2 enzymes were omitted from the reaction mixture. The reactions were terminated by adding 8 μl of 5x SDS-PAGE sample buffer and 4 μl of freshly prepared 1.0 M DTT. Reaction tubes were heated at 90 °C for 5 min and the samples were subjected to a 7.5% SDS-PAGE followed by Western blot assays.

### CHIP siRNA silencing

Cells grown on 60-mm culture dishes were transfected with 80 pmoles of CHIP-specific or scrambled stealth siRNAs (Ambion, ThermoFisher Scientific) using lipofectamine RNAiMAX reagent (Invitrogen). Cells were harvested at the indicated time points, lysed in RIPA buffer containing 1x protease inhibitor cocktail (Calbiochem, EMD-Millipore) and analyzed by SDS-PAGE/Western blot assays for the levels of CHIP and ALDH1L1 proteins. Experiments were repeated five times.

### Expression of CHIP in NIH3T3 cells

Cells were cultured in 35 mm plates and maintained on OptiMEM media supplemented with 10% FBS. Cells (80–90% confluent culture) were transfected with the pcDNA3-CHIP plasmid [23], 4 μg DNA per well, using Lipofectamine 3000 (ThermoFisher Scientific) according to the manufacturer’s instructions. Two independent experiments were performed.

### Immunoprecipitation

Cells were washed with ice-cold PBS and lysed in RIPA buffer, supplemented with protease inhibitor cocktail and phosphatase inhibitors, at 4 °C for 30 min. After centrifugation at 14000 x g for 15 min at 4 °C, supernatant (250 μg of total protein) was pre-cleared with protein G Sepharose 4 fast flow (GE Healthcare Life Sciences) for 1 h, and incubated with ALDH1L1-specific antibody (5 μg) overnight and then with 50 μl of protein G Sepharose 4 fast flow for an additional 3 h (all steps were carried out at 4 °C). Beads were pelleted and washed with cold PBS containing 1% NP-40 and 2 mM sodium orthovanadate. The pulled-down material was eluted with 100 mM glycine buffer, pH 3.0 and analyzed by SDS-PAGE/immunoblotting. Alternatively, ALDH1L1 was pulled down using Surebeads (BioRad) according to the manufacturer’s protocol. The HSP90 immunoprecipitation was carried out on protein A magnetic beads (New England Biolabs). Twenty-five μl of beads suspension was incubated with 200 μl crude cell extract at 4 °C for 1 h. Magnetic field was applied to pull beads to the side of the tube. Supernatant was transferred to a fresh tube and 5 μg anti-HSP90 antibody was added. The sample was vortexed, incubated at 4 °C for 1 h, and 25 μl of protein A magnetic beads suspension were added. The mixture was gently vortexed and incubated with agitation for 1 h at 4 °C. Magnetic field was applied to pull the beads to the side of the tube, and supernatant was discarded. The beads were washed twice with wash buffer (1x PBS + 0.1% Tween-20). Bead pellet was resuspended in 30 μl of 4x SDS sample loading buffer (Bio-Rad), and the samples were incubated at 70 °C for 5 minutes. Magnetic field was applied to the samples and the supernatant was loaded on SDS-PAGE.

### Deubiquitination of ALDH1L1

Endogenous ALDH1L1 pulled down from NIH3T3 cells was treated with 200 nM of catalytic domain of recombinant human USP2 or USP7 (Boston Biochem) at 37 °C for 1 h before SDS-PAGE/Western blot analysis.

### Assay of reduced folate pools

Folate pools were measured in cell lysates (obtained from approximately 5 x 10^6^ cells) by the ternary complex assay method as we previously described [5]. Folate levels were calculated per mg of cellular protein measured by Bradford assay. Experiments were performed three times with each sample done in quadruplicate.

## Results

### Levels of ALDH1L1 protein fluctuate through the cell cycle

**Fig 2:**
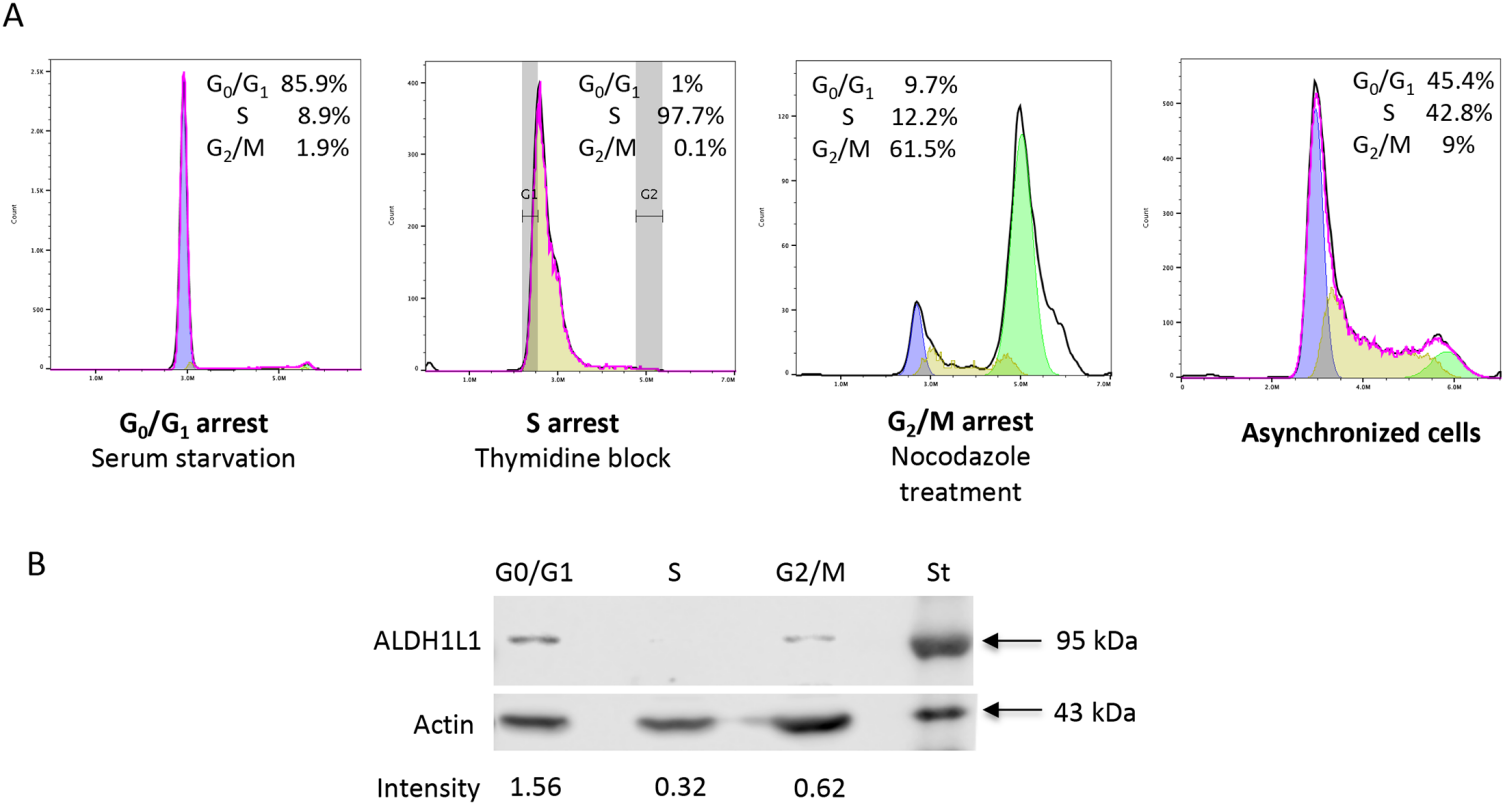
Levels of ALDH1L1 protein in NIH3T3 cells arrested at difference phases. **A**, NIH3T3 cells arrested in G_0_/G_1_ (serum starvation), S-phase (double thymidine block) or G_2_/M (double thymidine block and nocodazole treatment) phase. Asynchronous cells shown as a control. Numbers on the panels indicate distribution of cells between cell cycle phases. Fitted peaks are: *Blue*, calculated G_0_/G_1_ phase; *yellow*, S phase; *green*, G_2_/M phase. Cell cycle data were analyzed using FlowJo software. **B**, Western blot assay of ALDH1L1 in NIH3T3 cells arrested in indicated phase (20 μg of total cell lysate was loaded in each lane). Actin is shown as loading control. Arrows indicate molecular weight standards (St). Numbers show ALDH1L1 band intensity (arbitrary densitometry units) normalized to actin. Experiments were performed three times.

We evaluated levels of ALDH1L1 protein in NIH3T3 cells harvested in different stages of the cell cycle. We observed that ALDH1L1 levels were highest in confluent cells but immediately decreased after cells were split and plated to about 10–20% confluency (Fig 1B). Thirty-six hours after splitting, ALDH1L1 dropped to very low or undetectable levels indicating the loss of the protein in actively proliferating fibroblasts (Fig 1B and S1 Fig). Cell cycle analysis using the PI staining confirmed that at 36 h most cells were in the S-phase. In confluent culture, the majority of cells were in the G_0_/G_1_ phase, a typical stage for resting cells (Fig 1C). To investigate whether the ALDH1L1 down-regulation is S-phase specific, we arrested cells at G_0_/G_1_, S or G_2_/M phase and evaluated ALDH1L1 levels. Strong arrest in the corresponding phase was achieved in these experiments (Fig 2A). These experiments revealed that while ALDH1L1 was present in the G_0_/G_1_ and G_2_/M phases, the protein was lost in the S-phase (Fig 2B). Notably, the highest level of the protein was observed when cells became confluent and experienced the most contact inhibition and the least proliferation (Fig 1B).

### Interaction of ALDH1L1 with proteins in the ubiquitin-proteasome pathway

We used a pull-down approach in combination with LC/MS-MS analysis to identify ALDH1L1-interacting proteins. In this approach, pure human recombinant ALDH1L1 was loaded on the folate affinity column (5-formyl-THF covalently attached to agarose beads). In previous studies, 5-formyl-THF affinity columns were used to purify both endogenous and recombinant ALDH1L1 [20, 21, 24]. This enzyme tightly binds to 5-formyl-THF even in the presence of high salt (2 M KCl) and is eluted off the column only with high concentrations of folic acid (5–20 mM). We loaded the lysate of A549 cells (ALDH1L1-deficient cell line) on the column with bound ALDH1L1. The column was expected to retain ALDH1L1-interacting proteins. The ALDH1L1-interacting proteins were eluted by 2.0 M KCl. Prior to elution, the column was washed with loading buffer and 0.5 M KCl to ensure that only proteins tightly bound to ALDH1L1 were identified. In the control experiment, A549 cell lysate was passed through a column which was not pre-loaded with ALDH1L1. Washing and elution was performed as described above. Targets identified in the control experiments following the 2.0 M KCl elution were not considered as ALDH1L1 interacting proteins and were excluded from further analysis. Among identified ALDH1L1-interacting targets, a number of proteins were components of the ubiquitin-proteasome pathway including eight subunits of the proteasome, four ubiquitin hydrolases and the E3 ubiquitin-protein ligase CHIP (the carboxyl terminus of Hsp70-interacting protein, also known as STIP1 homology and U-box containing protein 1 or STUB1) (Table 1). These findings suggested that ALDH1L1 is a potential target for the proteasomal degradation. Our experiments have also identified HSP90 as a major ALDH1L1-interacting protein (Table 1).

**Table 1:** Proteins relevant to the ubiquitin-proteasome pathway pulled down on ALDH1L1.

Protein ID	Protein	Score*
IPI00414676.6	Heat shock protein HSP 90-beta	2650.57
IPI00784295.2	Isoform 1 of Heat shock protein HSP 90-alpha	2550.69
IPI00646721.1	Ubiquitin carboxyl-terminal hydrolase (USP7)	287.76
IPI00003964.4	Ubiquitin specific protease 9, X-linked isoform 4	206.37
IPI00184533.1	Ubiquitin carboxyl-terminal hydrolase 11	40.70
IPI00165528.1	Isoform 2 of Ubiquitin carboxyl-terminal hydrolase 47	15.87
IPI00645380.1	Isoform 2 of STIP1 homology and U box-containing protein 1 (STUB1, E3 ubiquitin-protein ligase CHIP)	16.73
IPI00216770.1	26S protease regulatory subunit 6B Isoform 2	73.62
IPI00024821.1	26S proteasome non-ATPase regulatory subunit 14	63.98
IPI00021435.3	26S protease regulatory subunit 7	36.56
IPI00018398.4	26S protease regulatory subunit 6A	30.05
IPI00456695.1	26S proteasome non-ATPase regulatory subunit 1, Isoform 2	25.00
IPI00555749.1	26S proteasome ATPase subunit 5 variant	23.48
IPI00185374.4	26S proteasome non-ATPase regulatory subunit 12	23.26
IPI00011126.6	26S protease regulatory subunit 4	17.47

*Score, abundance of proteins in the ALDH1L1 pull-down based on the intensity of peptide peaks from LC/MS-MS detection.

### ALDH1L1 is a target for proteasomal degradation

**Fig 3:**
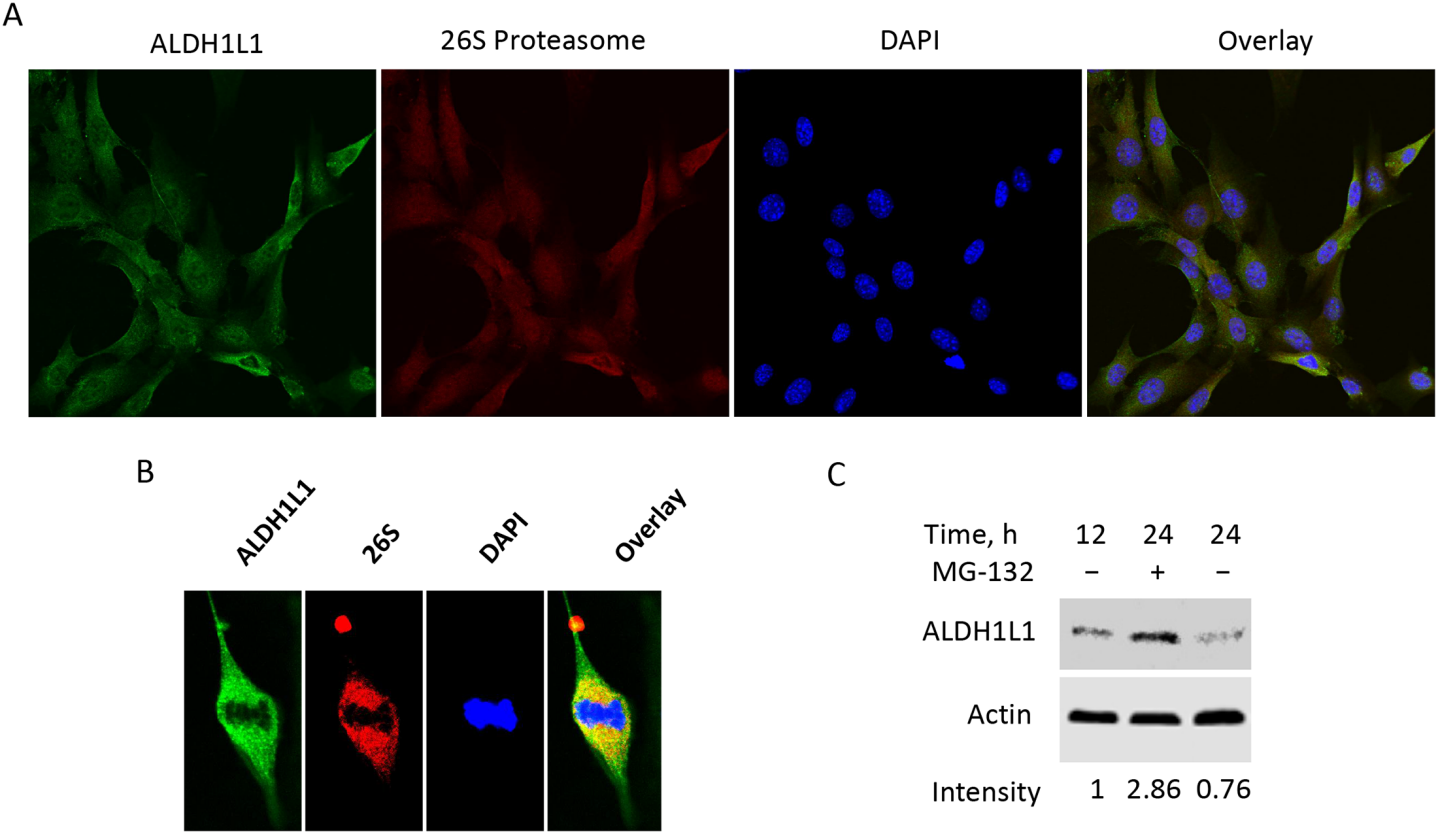
ALDH1L1 is a target of proteasomal degradation. **A**, confocal microscopy of NIH3T3 cells stained with ALDH1L1-specific antibody/secondary anti-rabbit antibody conjugated with Alexa Fluor 488 dye and 26S proteasome-specific antibody (against S2 protein)/secondary anti-mouse antibody conjugated with Alexa Fluor 555 dye; cells were also counterstained with DAPI. **B**, a single cell from *A* at higher magnification. **C**, proteasome inhibitor MG-132 protects ALDH1L1 from degradation (after splitting confluent culture, cells grew for 12 h before addition of MG-132 (5 μM); 12 h later, cells were subjected to ALDH1L1 Western blot assay; time after splitting confluent culture is indicated. Quantification of ALDH1L1 bands (arbitrary densitometry units) normalized to actin is indicated. This experiment was repeated four times.

If ALDH1L1 is a target for proteasomal degradation, it is expected that the protein will co-localize with proteasomes. To address this matter, we used confocal microscopy of fixed NIH3T3 cells to visualize ALDH1L1 and proteasomes. To perform two color staining, mouse monoclonal antibody against 26S proteasome subunit A2 in combination with goat anti-mouse secondary antibody conjugated with Alexa Fluor 555 dye, and rabbit polyclonal ALDH1L1 antibody in combination with secondary chicken anti-rabbit antibody conjugated with Alexa Fluor 488 dye, were used. These experiments show the co-localization of ALDH1L1 with proteasomes (Fig 3A and 3B). To further investigate the involvement of proteasomal pathway in ALDH1L1 degradation, we treated NIH3T3 cells with the proteasomal inhibitor MG-132 [25]. MG-132 (5 μM) prevents the decrease of ALDH1L1 in actively proliferating cells (Fig 3C), supporting the role for proteasomal pathway in the ALDH1L1 degradation.

### *In vivo* ubiquitination of ALDH1L1

**Fig 4:**
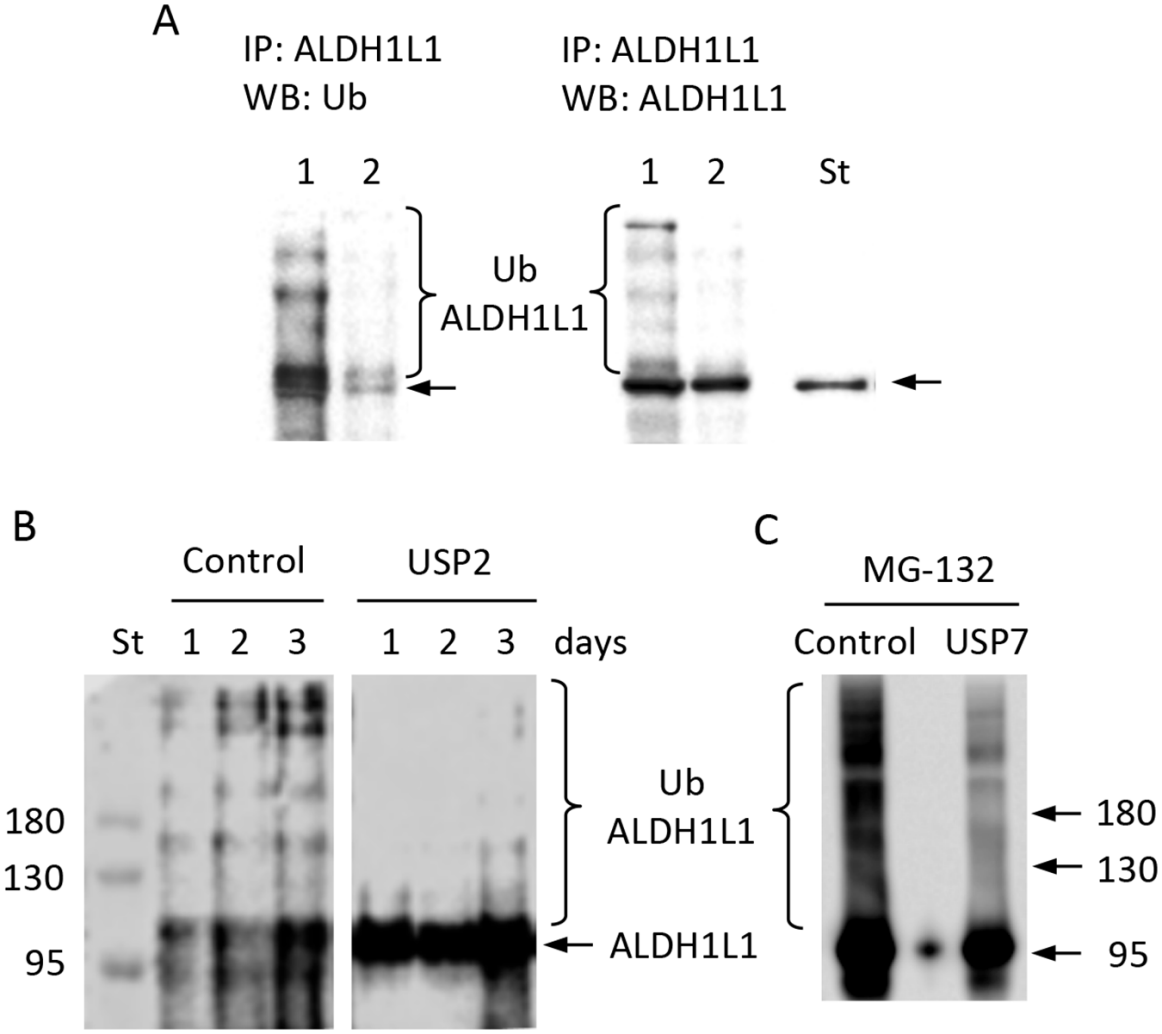
ALDH1L1 is ubiquitinated in NIH3T3 cells. **A**, ALDH1L1 pulled-down from NIH3T3 cell lysates using ALDH1L1-specific antibody and protein A beads; elution with glycine buffer (*lane 1*), followed by elution with SDS-PAGE loading buffer (*lane 2*). Proteins were resolved on a 7.5% SDS-PAGE gel followed by Western blot assay with ubiquitin-specific antibody (*left panel*) or ALDH1L1-specific antibody (*right panel*). Lane *St* is purified recombinant ALDH1L1. **B**, ALDH1L1 was immunoprecipitated from NIH3T3 cell lysates using an ALDH1L1-specific antibody and Protein A Magnetic beads; samples were resolved on a 7.5% SDS-PAGE followed by Western blot assay with anti-ubiquitin monoclonal antibody. Cells were harvested at different time points after splitting (as indicated); lysates were treated with deubiquitinase inhibitor (4.0 μM recombinant human ubiquitin aldehyde C-terminal derivative) prior to immunoprecipitation. After immunoprecipitation, eluates were treated with deubiquitinase (200 nM of recombinant human USP2 catalytic domain); *control*, untreated lysates. **C**, ALDH1L1 was immunoprecipitated from NIH3T3 cells as in **B** and treated with USP7. Cells were treated with 10 μM MG-132 for 4 h before the pull-down. After treatment with USP7, we have repeated the pull-down with ALDH1L1-specific antibody and detected ubiquitinated species as in **B**.

The canonical proteasomal pathway requires protein ubiquitination prior to degradation. Some proteins, however, can undergo proteasomal degradation without ubiquitination [26, 27]. Bioinformatics analysis using several online tools (BDM-PUB: Prediction of Ubiquitination sites with Bayesian Discriminant Method; UbPred: predictor of protein ubiquitination sites, UCSD; UbiSite, http://csb.cse.yzu.edu.tw/UbiSite/) indicated the presence of putative ubiquitination sites in ALDH1L1. To determine whether ALDH1L1 is ubiquitinated before the proteasomal degradation, we used ALDH1L1-specific antibody to pull-down ALDH1L1 from NIH3T3 fibroblasts. Western blot assays of immunoprecipitated samples using ALDH1L1-specific or ubiquitin-specific antibody visualized protein bands with molecular masses above 99 kDa. These bands correspond to a covalently modified ALDH1L1 and are consistent with the protein ubiquitination (Fig 4A). To further confirm that the observed bands represent ubiquitinated ALDH1L1, the pulled-down samples were treated with deubiquitinase, USP2 (ubiquitin carboxyl-terminal hydrolase 2; 200 nM of recombinant human USP2 catalytic domain). To prevent the loss of ALDH1L1-bound ubiquitin due to activities of endogenous hydrolases, cell lysates were treated with 4.0 μM recombinant human ubiquitin aldehyde C-terminal derivative, a deubiquitinase inhibitor, prior to the immunoprecipitation. Similar to previous experiments, we observed bands with molecular mass higher than ALDH1L1 (Fig 4B) in the absence of USP2. The treatment of the lysates with USP2 resulted in the complete disappearance of bands with molecular mass higher that 99 kDa (Fig 4B), indicating ALDH1L1 is ubiquitinated in NIH3T3 cells. We repeated this experiment using USP7, the ubiquitin carboxyl-terminal hydrolase most abundant in the ALDH1L1 pull-down (Table 1). In this specific experiment, NIH3T3 cells were treated for 4 h with MG-132 (10 μM) before the pull-down with the ALDH1L1-specific antibody to prevent the proteasomal degradation. We observed that ALDH1L1 was heavily ubiquitinated in this pulldown, whereas the treatment with USP7 strongly decreased the presence of ubiquitinated species (Fig 4C).

### Purified recombinant ALDH1L1 is ubiquitinated in vitro by the E3 ligase CHIP

**Fig 5:**
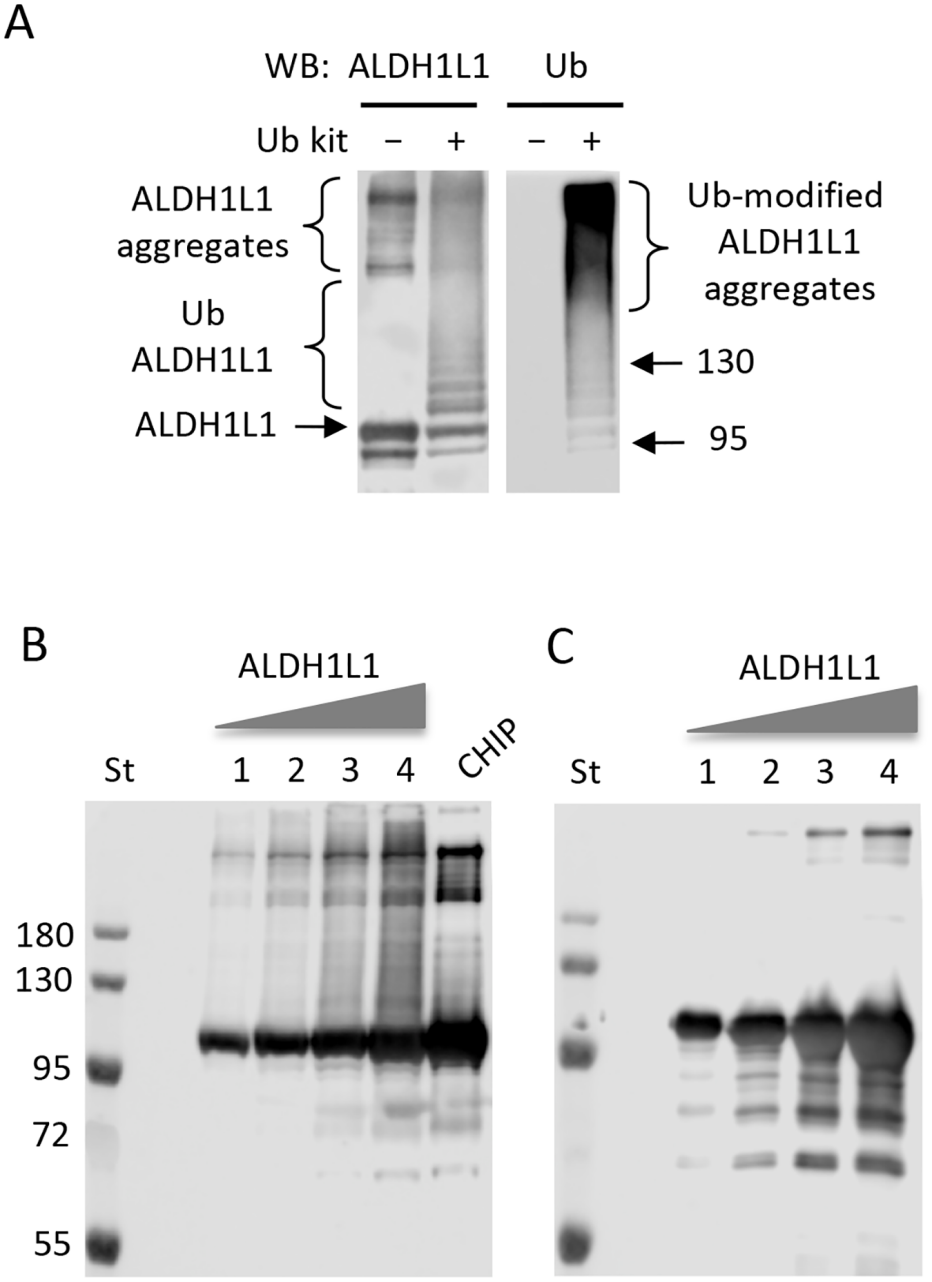
*In vitro* ubiquitination of purified ALDH1L1. **A**, purified ALDH1L1 (1.1 μg) was ubiquitinated using the *in vitro* ubiquitination kit that included CHIP E3 ligase (see Materials and methods). Reaction products were resolved by 7.5% SDS-PAGE followed by Western blot assay with ALDH1L1-specific antibody (*left panel*) or Ub-specific antibody (*right panel*). *Arrows* indicate positions of 95 kDa and 130 kDa pre-stained molecular mass protein standards. **B**, lanes 1–4, increased amount of ALDH1L1 (0.6, 1.2, 2.4 and 4.8 μg) were subjected to *in vitro* ubiquitination with NIH3T3 cell lysate; ubiquitination with CHIP was the positive control. **C**, negative control for panel **B**, lanes 1–4, untreated purified ALDH1L1 (0.6, 1.2, 2.4 and 4.8 μg) prior to ubiquitination. Each experiment was repeated at least three times. Molecular mass standards (*St*) are the same for all panels.

Protein ubiquitination requires the participation of specific enzymes, E3 ligases [28]. Based on the analysis of targets identified as ALDH1L1-interacting proteins (Table 1), we proposed CHIP (STUB1) [29–31] as the putative E3 ligase, that ubiquitinates ALDH1L1. Here we showed that CHIP ubiquitinates ALDH1L1 *in vitro*: the addition of recombinant CHIP to recombinant ALDH1L1 in the presence of other ubiquitination system components (E1 and E2 enzymes, ubiquitin and ATP) leads to the formation of covalent ALDH1L1-ubiquitin complexes (Fig 5A). The staining of the blot with an anti-ubiquitin antibody confirmed that the laddering above ALDH1L1 band (99 kDa) is associated with the ALDH1L1 ubiquitination (Fig 5A). Of note, stronger ubiquitin signal (Fig 5A) was seen for high molecular mass bands (above 180 kDa). It should be noted that ALDH1L1 exists as a tetramer of identical subunits, and it does not dissociate to monomers or dimers under standard physiological condition but apparently can form bigger aggregates [32]. Upon ubiquitination, these aggregates will have higher number of ubiquitin molecules, which is reflected in the intensity of the signal in Fig 5A. It is not clear at present whether the ubiquitination promotes aggregation or stabilizes existing aggregates.

**Fig 6:**
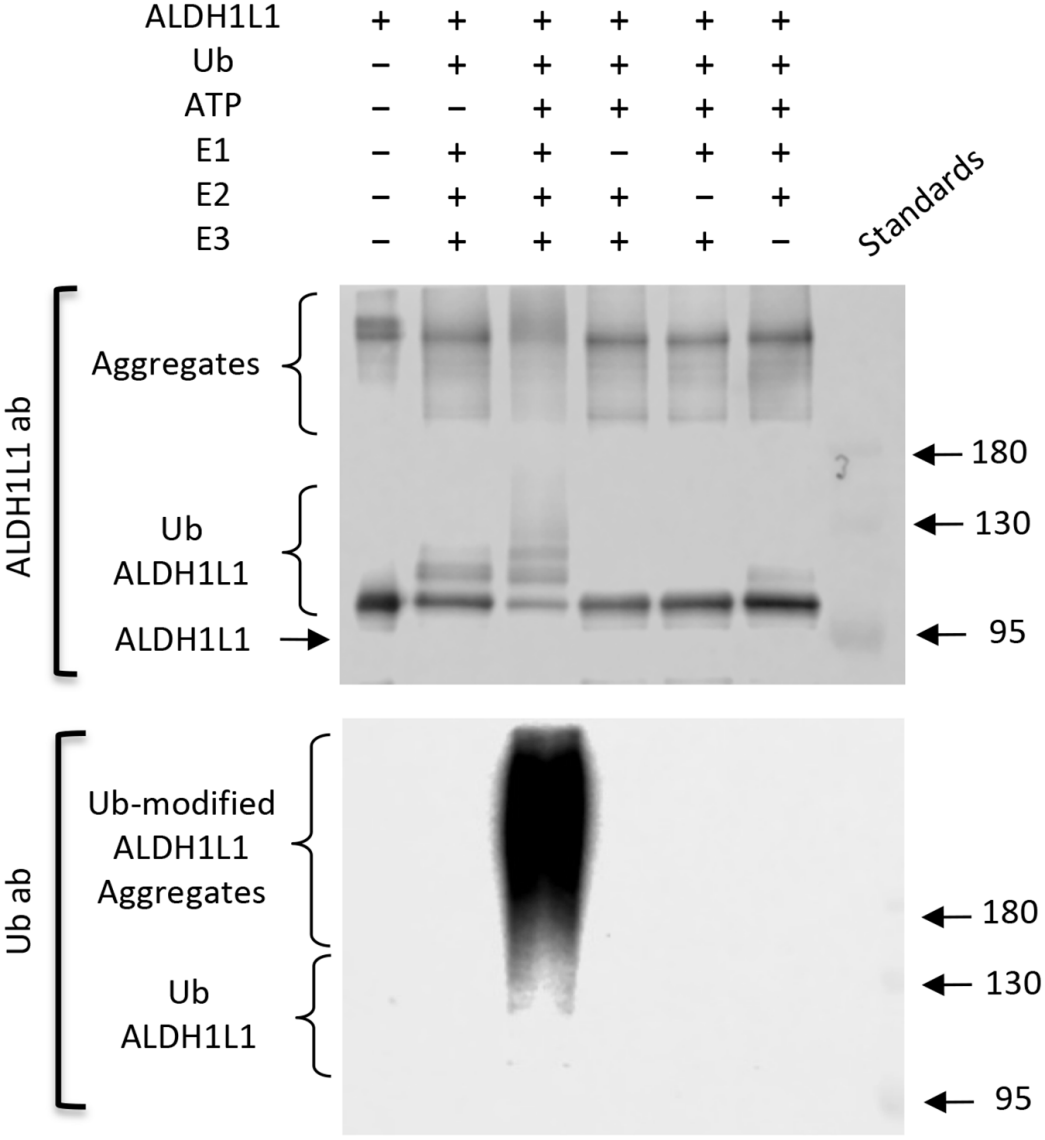
Omitting components of ubiquitination machinery prevents *in vitro* ALDH1L1 ubiquitination. Purified ALDH1L1 (1.1 μg) was incubated for 1 h with the *in vitro* ubiquitination kit that included all components or with omission of ATP, E1, E2 or E3 (see Materials and methods). Reaction products were resolved by 7.5% SDS-PAGE followed by Western blot assay with ALDH1L1-specific antibody and Ubiquitin (Ub)-specific antibody. *St*, pre-stained molecular mass protein standards (numbers on the right indicates standard molecular masses, kDa). Experiment was performed three times with the same outcome.

In a similar experimental setting, we evaluated if the NIH3T3 cell lysate has the potential to ubiquitinate recombinant ALDH1L1. Protein bands corresponding to ubiquitin-conjugated ALDH1L1 were observed following the incubation with cell lysates (Fig 5B). This pattern coincides with the band pattern observed in the experiment with CHIP as the positive control (Fig 5B), indicating that the lysate was efficient in the *in vitro* ubiquitination of ALDH1L1. Control reactions, where cell lysate was omitted, did not generate ALDH1L1-ubiquitin bands (Fig 5C). Similarly, *in vitro* reconstitution experiments with ALDH1L1 and all components of the ubiquitination system resulted in the polyubiquitination of ALDH1L1 (Fig 6, *upper panel*). To confirm that the modifications were due to addition of ubiquitin, blots were stripped and reprobed with anti-ubiquitin antibodies (Fig 6 *lower panel*). Omitting ATP, E1 or E2 enzymes from the *in vitro* reaction prevented ALDH1L1 ubiquitination indicating that non-specific interactions of CHIP or ubiquitin are not responsible for the ubiquination of ALDH1L1 (Fig 6).

### ALDH1L1 interacts with CHIP and CHIP-assisting chaperone HSP90 *in vivo*

**Fig 7:**
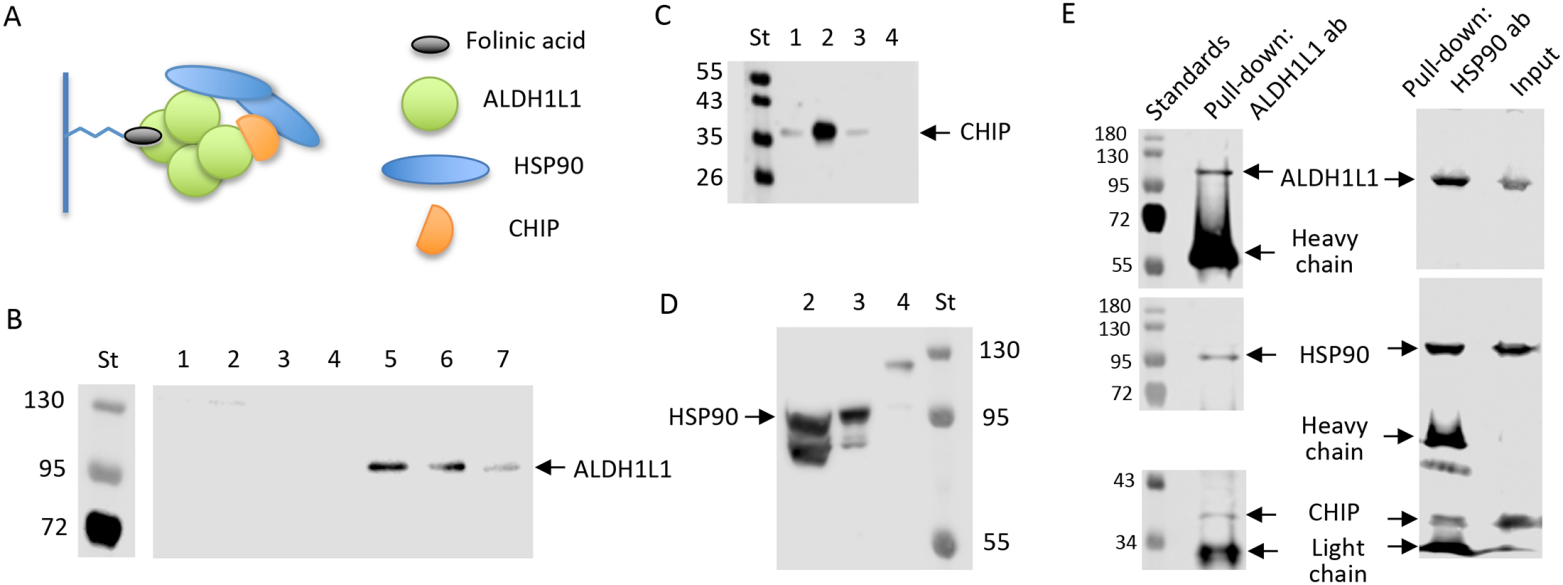
ALDH1L1 interacts with E3 ligase CHIP and CHIP-assisting chaperone HSP90 *in vivo*. **A**, schematic depicting the pull-down assay on a folate affinity column. **B**, endogenous ALDH1L1 interacts strongly with immobilized folinic acid (after loading NIH3T3 cell lysate on the affinity column, the following eluted fractions were collected and analyzed by SDS-PAGE/Western blot assay with ALDH1L1-specific antibody: *lane 1*, washing buffer; *lane 2*, 0.5 M KCl; *lane 3*, 1.0 M KCl; *lane 4*, 2.0 KCl; *lane 5*, 5 mM folic acid in 2.0 KCl; *lane 6*, 20 mM folic acid in 2.0 M KCl; *lane 7*, purified ALDH1L1 standard; *St*, molecular masses standards (indicated by numbers in kDa on the *left*). **C**, samples as in **B** probed with CHIP-specific antibody. **D**, samples as in *B* probed with HSP90-specific antibody. **E**, Immunoprecipitation of NIH3T3 cell lysate with ALDH1L1-specific (*left panels*) or HSP90-specific (*right panels*) antibody followed by SDS-PAGE/Western blot assay with ALDH1L1-specific, HSP90-specific and CHIP-specific antibodies. In each experiment (ALDH1L1 or HSP90 pull-down) the same blot was stripped twice and reprobed. Numbers indicate molecular masses (kDa) for standards.

We used folate-affinity chromatography to investigate the interaction of ALDH1L1 with the E3 ubiquitin-protein ligase CHIP in NIH3T3 cells (Fig 7A). As expected, ALDH1L1 present in these cells was retained by the folate-affinity column and eluted with 5 mM and 20 mM folic acid (Fig 7B). Fractions eluted from the column were also probed with a CHIP-specific antibody. The CHIP protein was retained by the folate column and eluted with 0.5–1.0 M KCl (Fig 7C, lane 2), indicating strong interaction with ALDH1L1. It is not clear whether CHIP interacts directly with ALDH1L1 or if the interaction requires a scaffold protein. Previous reports described the scaffolding function of HSP90 for CHIP acting as E3 ligase on HSP90-client proteins [33–35]. Since HSP90 was identified as an interacting partner of ALDH1L1 (Table 1), it may serve as the scaffold for CHIP and ALDH1L1. In support of this role, HSP90 was co-purified with ALDH1L1 and CHIP on folate-affinity column (Fig 7D). Immunoprecipitation of NIH3T3 cell lysate with ALDH1L1-specific antibody also co-immunoprecipitated HSP90 and CHIP (Fig 7E). In turn, the pull-down with HSP90-specific antibody co-immunoprecipitated ALDH1L1 and CHIP (Fig 7E). These data indicate that all three proteins form a complex in NIH3T3 cells.

### Degradation of ALDH1L1 in NIH3T3 cells is CHIP-dependent

**Fig 8:**
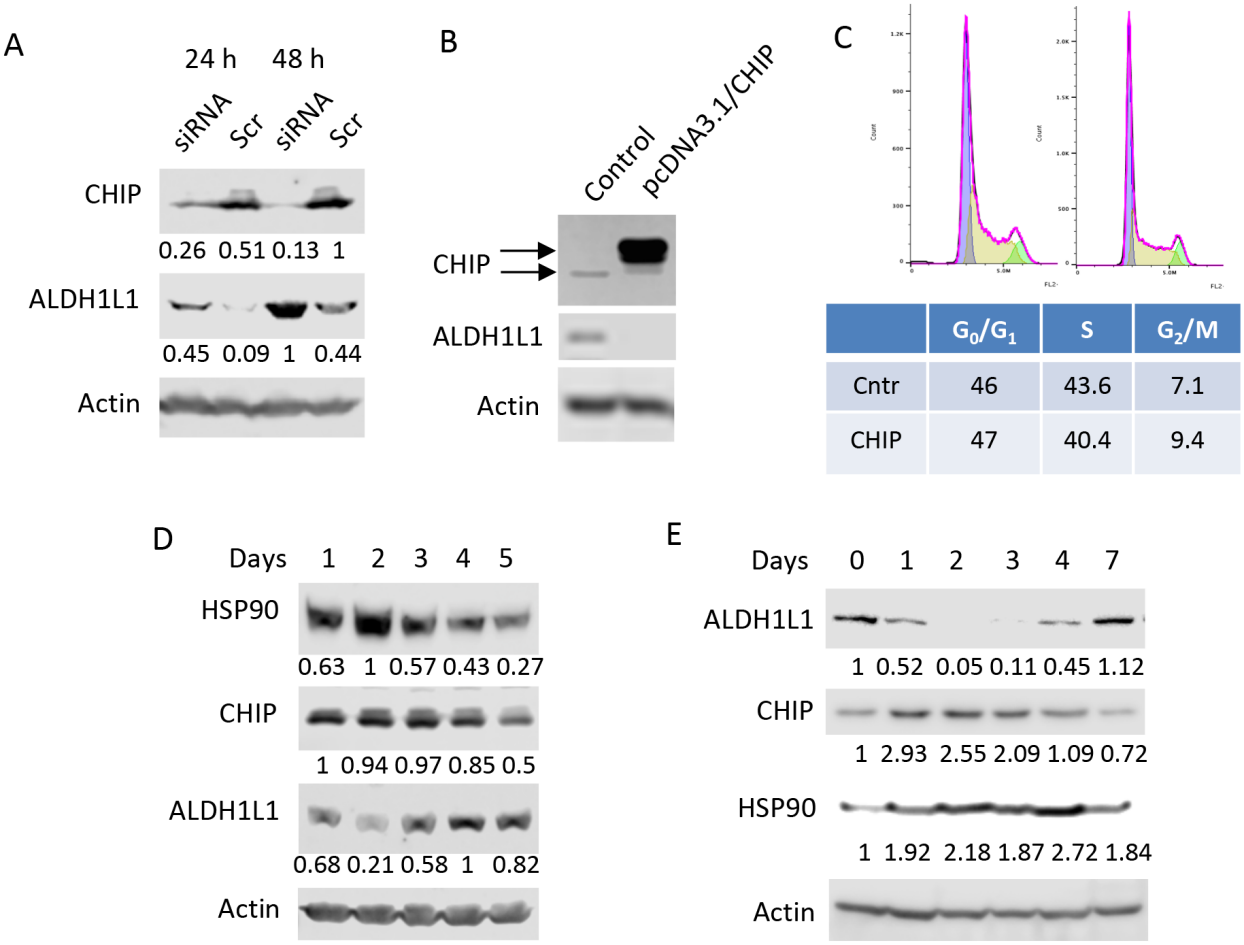
CHIP is required for the ALDH1L1 degradation. **A**, levels of CHIP and ALDH1L1 (Western blot assay) in NIH3T3 cells transfected with CHIP siRNA (*siRNA*) or scrambled siRNA (*Scr*); experiments were repeated six times. **B**, forced expression of CHIP in NIH3T3 cells leads to the loss of ALDH1L1. Arrows indicate endogenous and transiently expressed CHIP (the latter has a higher molecular mass due to the presence of His-tag and FLAG-tag). **C**, overexpression of CHIP in NIH3T3 cells does not affect the cell cycle. Cell cycle distribution of asynchronous NIH3T3 cells transfected with "empty plasmid" (control), or CHIP expression vector did not show statistically significant differences. Experiments were performed in triplicate and the expression of CHIP was verified by Western blot. Levels (Western blot assays) of CHIP and HSP90 inversely correlate with levels of ALDH1L1 during proliferation of NIH3T3 (**D**) or AML12 (**E**) cells. In panels **A**, **B**, **D** and **E** actin is shown as loading control. Quantification of corresponding protein bands (arbitrary densitometry units) normalized to actin is indicated.

To investigate the role of CHIP in the ALDH1L1 regulation, we knocked down the ligase in NIH3T3 cells using CHIP-specific siRNA duplex and evaluated levels of ALDH1L1. The CHIP protein levels significantly decreased 24 h post-siRNA transfection with strong silencing achieved 48 h post-transfection (Fig 8A). Simultaneously with the decrease in the CHIP protein, we also observed elevation of ALDH1L1 (Fig 8A). To further confirm the role of CHIP in the ALDH1L1 degradation, we have expressed it in NIH3T3 cells through a transient transfection. Strong expression of CHIP was achieved in this experiment (Fig 8B). This experiment demonstrated that CHIP expression lead to the complete loss of ALDH1L1 (Fig 8B). Of note, the CHIP expression did not perturb the cell cycle in NIH3T3 cells: the distribution of asynchronous cells between phases was almost identical between control and CHIP-expressing cells (Fig 8C). Interestingly, levels of HSP90 and CHIP in NIH3T3 cells inversely correlated with levels of ALDH1L1 (Fig 8D). We have also evaluated levels of ALDH1L1 in murine hepatocyte cell line, AML12, at different time points following splitting of a confluent AML12 culture. As in the case of NIH3T3 cells, levels of ALDH1L1 fluctuated in AML12 cells being lowest in actively proliferating cells (days 2 and 3 after splitting) and highest in the fully confluent culture (day 7) (Fig 8E). Likewise, inverse correlation between levels of ALDH1L1 and levels of CHIP or HSP90 was observed in AML12 cells (Fig 8E).

### Alterations of reduced folates during the cell cycle progression

**Fig 9:**
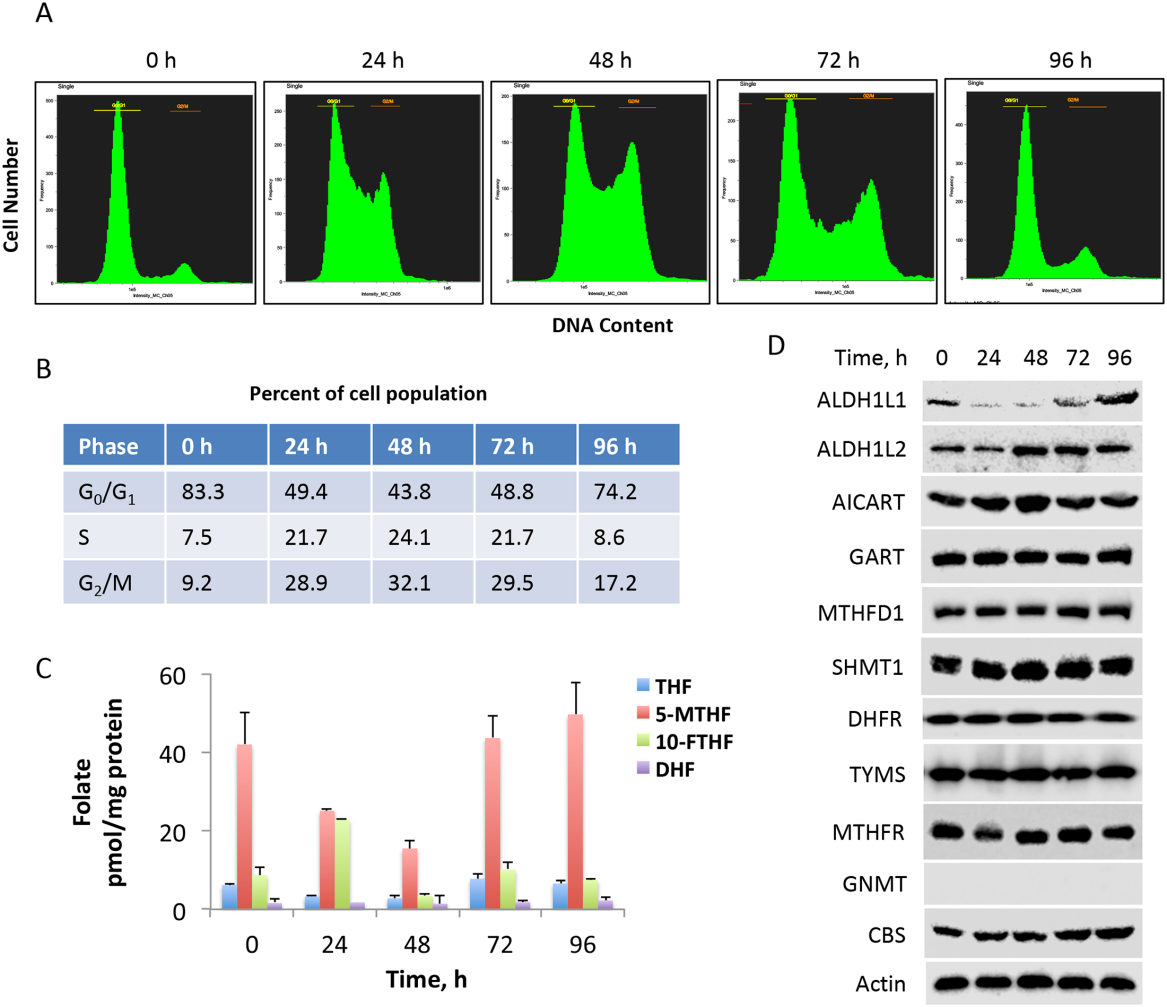
Levels of reduced folate coenzymes fluctuate with cell cycle progression in NIH3T3 cells. **A**, distribution of NIH3T3 cells between cell cycle phases at different time points after splitting confluent cell cultures (release from contact inhibition). **B**, percent cell population in G_0_/G_1_, S, and G_2_/M phases of the cell cycle (calculated from *A*). **C**, levels of folate coenzymes at different time points of cell culture (time points correspond to those in panel *A*); THF, tetrahydrofolate (the data represent combined tetrahydrofolate and 5,10-methylenetetrahydrofolate pools); 5-MTHF, 5-methyltetrahydrofolate; 10-FTHF, 10-formyltetrahydrofolate; DHF, dihydrofolate (the data represent combined DHF and folic acid pools). **D**, levels of folate-related enzymes during the progression through the cell cycle of NIH3T3 cells.

Cells maintain several forms of reduced folate coenzymes which differ by the oxidation level of bound one-carbon group. The ternary complex assay used in our study measures 5-methyl-THF, 10-formyl-THF, combined THF/5,10-methylene-THF and combined DHF/FA pools [5, 36]. Because this assay does not discriminate between forms of folate coenzymes with different number of glutamic acid residues, the readout for each coenzyme form is the total pool including all differentially polyglutamylated molecules. We have evaluated the reduced folate pools in NIH3T3 cells progressing from active proliferation to quiescent state. Between 24 h and 72 h, the highest proportion of cells was in S-phase (Fig 9A and 9B). At 0 h (immediately after splitting) and 96 h, cells were in G_0_/G_1_-phase (Fig 9A and 9B). We have found that the predominant folate form in NIH3T3 fibroblasts in all states is 5-methyl-THF (Fig 9C), which is the prevalent pool in many cell types. We have further observed that at 48 h, when NIH3T3 cells most actively proliferate, all folate pools are significantly decreased (Fig 9C). Notably, at 24 h when the cell culture shifts from quiescent to actively proliferating, the levels of the ALDH1L1 substrate 10-formyl-THF are strongly increased, reaching the level of 5-methyl-THF, which in turn is significantly decreased (Fig 9C). Remarkably, 10-formyl-THF is the only folate coenzyme that shows this trend. As the NIH3T3 cell culture becomes more confluent, levels of folate coenzymes reach levels observed in resting cells (Fig 9C). We have also evaluated levels of several enzymes of folate metabolism in NIH3T3 cells progressing from proliferation to quiescence. In contrast to ALDH1L1, tested enzymes remained fairly constant through the cell cycle (Fig 9D). Interestingly, ALDH1L2, the mitochondrial homolog of ALDH1L1 [18], has shown rather opposite regulation during the cell cycle being slightly elevated in proliferating cells (Fig 9D). In contrast to ALDH1L1, GNMT, another folate-related enzyme commonly silenced in human cancers [17], was not expressed in NIH3T3 cells (Fig 9D).

## Discussion

In the present study, we demonstrate that levels of ALDH1L1 in two cell lines, NIH3T3 and AML12, are tightly regulated during cell cycle progression. The highest enzyme levels are observed in quiescent cells, which have exited the cell cycle due to the contact inhibition [37]. After passaging as cells reentered the cell cycle following density reduction, ALDH1L1 protein levels drop dramatically. The change in the protein expression profile during the cell progression through the cell cycle is well known phenomenon associated with the precise control of the DNA replication [38]. In addition to cell cycle checkpoint-controlling proteins, enhanced expression of proteins involved in biosynthetic pathways (i.e. nucleotide synthesis) supporting DNA replication is required as cells enter and progress through S-phase [39, 40]. Folate enzymes supporting nucleotide biosynthesis, a key pathway activated in rapidly proliferating cell, are commonly elevated in S-phase [41–45]. In contrast, ALDH1L1, which directly competes with the *de novo* purine biosynthesis for the same substrate, 10-formyl-THF, was downregulated (Fig 1A and 1B). The decrease of cellular 10-formyl-THF by ALDH1L1 restricts the capacity of the cell to produce purines [4, 36]. Perhaps as an alternative to the elevation of biosynthetic enzymes [5], which are already expressed at high levels, the shutdown of the restrictor is implemented as a mechanism to maintain enhanced purine biosynthesis. ALDH1L1 not only competes with the *de novo* purine pathway for the same substrate, 10-formyl-THF, but also depletes one-carbon groups from the folate pool by converting folate-bound formyl to CO_2_, potentially affecting other folate-dependent biosynthetic pathways. While the finding that ALDH1L1 is down regulated in S-phase agrees with the anti-proliferative function of the enzyme [7], the mechanism of such robust effect on the protein was not clear.

Previously, we reported that the common mechanism of ALDH1L1 down-regulation in cancer cells is its promoter methylation [10]. This mechanism results in a sustained gene silencing and is not typically involved in the cell cycle regulation [46]. During a cell cycle transition, a main mechanism of altered gene expression is transcriptional regulation executed by specific transcription factors [47, 48]. While this type of regulation was not demonstrated for the *Aldh1l1* gene, during early mouse brain development ALDH1L1 protein expression inversely correlates with the transcription factor PAX3 [15]. These data imply that PAX3 might negatively regulate *Aldh1l1* transcription. However, the rapid drop in ALDH1L1 levels during the G_0_ to S-phase transition observed in our study cannot be attributed to the loss of its mRNA and likely involves protein degradation mechanisms. Two principal degradation pathways are involved in cellular protein turnover, lysosomal/autophagosomal and proteasomal [49]. The latter is a common pathway for cell cycle-specific protein clearance [38, 50]. Several enzymes of folate metabolism have been shown to undergo proteasomal degradation [43, 51–53]. In the present study, we identified numerous subunits of the 26S proteasome, as well as CHIP E3 ligase, as interacting partners of ALDH1L1. This finding suggests that the ubiquitin-proteasome pathway is involved in the rapid clearance of ALDH1L1. In further support of this mechanism of the regulation of ALDH1L1 levels, we have demonstrated that the treatment of NIH3T3 cells with proteasome inhibitor MG-132 inhibits clearance of the protein.

The global characterization of the human ubiquitin-modified proteome by the antibody-based capture of tryptic digest generating diglycine-containing isopeptides, a signature of ubiquitination, identified ~19,000 modified lysine residues within ~5000 proteins [54]. Among these proteins, there are several enzymes of folate pathways including SHMT1, SHMT2, MTHFD1, MTHFD2, GART, ATIC, MTRR, and DHFR. Interestingly, this search has also identified thymidylate synthase as a protein with 8 ubiquitination sites [54], though previously this protein was characterized as a target for proteasomal degradation in the absence of this modification [53]. While ALDH1L1 was not identified by the analysis as a target for ubiquitination, the study was carried out in HCT116 cells which are ALDH1L1 deficient due to gene silencing [10]. Of note, computational prediction of ubiquitination sites using several online tools indicated presence of numerous potential ubiquitination sites which overlap in mouse and human ALDH1L1. Interestingly, ALDH1L1 is a target for modification by the ubiquitin-like modifier MNSF-ß, which attaches at Lys72 [55]. While the function of this modification is not clear, it may regulate apoptosis in thymocytes [55].

The key components of the ubiquitin-proteasome degradation pathway that selectively recognize protein substrates are E3 ligases [50]. More than 600 E3 ligases are identified in the human genome with many of them demonstrating significant redundancy towards their substrates [28]. The E3 ligase CHIP, a key protein involved in ALDH1L1 degradation, has multiple validated targets [31]. CHIP is a master regulator of proteasomal protein degradation via chaperones and is involved in many biological processes [31]. The protein has dual functions, one as a co-chaperone of Hsp70 and Hsp90, and the other as an E3 ubiquitin ligase that mediates proteasomal degradation of chaperone client proteins [31]. CHIP has also the ability to polyubiquitinate protein targets, thus functioning as an E4 ligase [30]. For some HSP90 client proteins, the function of CHIP is redundant with the function of other E3 ligases [56]. This, however, was not the case for ALDH1L1: CHIP siRNA silencing prevented ALDH1L1 degradation in our experiments. In agreement with this finding, overexpression of CHIP in NIH3T3 cells resulted in the total loss of ALDH1L1. The role of E3 ubiquitin ligases in the regulation of the cell cycle progression is well appreciated [50]. The E3 ligases are involved in degradation of cyclins, Cdc25 phosphatases, Polo-like kinases as well as CDK inhibitors such as p21/Waf1 [50]. Likewise, CHIP was linked to the cell cycle progression: CHIP down regulation by miR-1178 affects the G_1_/S phase transition in pancreatic cancer cells [57]. Furthermore, CHIP targets including p53 tumor suppressor have roles in cell cycle progression and cellular proliferation [31]. Overall, CHIP participates in protein turnover [29], which is in line with our findings of the CHIP-mediated ALDH1L1 degradation during the cell cycle.

Our study suggests that CHIP-mediated ALDH1L1 degradation involves the heat shock protein, HSP90. HSP90, a major member of the heat shock protein family, is a molecular chaperone regulating stability and activity of many proteins [58, 59]. HSP90 generally stabilizes its client proteins but it can also promote protein degradation by cooperating with the ubiquitin-proteasome system [58]. Though proteasomal degradation often involves incorrectly folded proteins, it is also important for the degradation of functional proteins when their levels need to be temporally regulated [60, 61]. In this regard, HSP90 preferentially binds to proteins completing their folding or to already folded functional proteins [62, 63]. HSP90 also binds locally structured regions in globally unfolded proteins [64]. In cooperation with HSPs, CHIP ubiquitinates unfolded proteins [31] but it is not clear whether it can ubiquitinate folded proteins. Toward this end, the question of whether ALDH1L1 degradation during the cell cycle transition involves functional protein or unfolded/aggregated protein remains obscure. Though the functional recombinant ALDH1L1 was a good target for ubiquitination by the CHIP/HSP complex in our *in vitro* experiments, the unfolding or aggregation of ALDH1L1 caused by its interaction with HSP90 cannot be excluded as a mechanism initiating the degradation. Whether additional proteins are required for the ALDH1L1 degradation is also an open question. Binding of some cell cycle regulating proteins to the HSP90/CHIP/ALDH1L1 complex may act as a signal initiating ALDH1L1 degradation at the transition from G_1_ to S-phase. We suggest that the CHIP-mediated ubiquitin-proteasome degradation of ALDH1L1 is involved in the protein regulation during the embryonic development when its rapid but temporal clearance is needed to enable proper cellular proliferation.

## Supporting information

S1 Table List of primary antibodies used in this study for Western blot assays. (PDF)

S1 Fig Relative content of ALDH1L1 protein in NIH3T3 cells from quantification of Western blot bands (normalized to actin; representative image is shown in Fig 1B). Samples for the assay were prepared from cells collected at indicated time points (hours) after splitting the culture. Average of three independent experiments and standard errors are shown. This experiment was repeated multiple times (more than ten experiments total) with essentially the same outcome.
(PDF)

S2 Fig Western blots of NIH3T3 cells used to create Fig 8D of the main manuscript. SDS-PAGE (7, 10 or 12% gels were used depending on the target) was followed by Western blot assays with specific antibody. Equal amount of cell lysates (20 μg of total protein) was loaded in each case. Membranes were stripped and re-probed for actin (not shown) to quantify each band relative to actin (S3 Fig). Samples were prepared from NIH3T3 cells collected at different times after splitting (reflects different confluence and the distribution of cells between cell cycle phases, Figs 1 and 8A and 8B). Lane 1, 0 h; lane 2, 24 h; lane 3, 48 h; lane 4, 72 h; lane 5, 96 h (the extra lane in the AICAR blot marked by asterisk corresponds to 120 h). All in-house polyclonal antibodies were verified in previous publications as indicated in Materials and Methods and S1 Table.
(PDF)

S3 Fig Plotted values from quantification of bands (normalized to actin) from S2 Fig (also Fig 9D in the main manuscript). Samples for the assay were prepared from cells collected at indicated time points (hours) after splitting the culture.
(PDF)
