# Supplementary material for: CHIP E3 ligase mediates proteasomal degradation of the proliferation regulatory protein ALDH1L1 during the transition of NIH3T3 fibroblasts from G0/G1 to S-phase
Source: PLoS One. 2018 Jul 6;13(7):e0199699. doi: 10.1371/journal.pone.0199699 (PMC6034817; doi:10.1371/journal.pone.0199699)
Supplement: S1 Table — (PDF) [file pone.0199699.s001.pdf]

S1 Table. List of primary antibodies used in this study for Western blot assays.

Target	Antibody	Company	Cat # or reference*	Dilution
AICART	Rabbit polyclonal	In-house	[5]	1:10,000
ALDH1L1	Rabbit polyclonal	In-house	[5,7,16,18]	1:10,000
ALDH1L2	Rabbit polyclonal	In-house	[18]	1:10,000
β-Actin	Mouse monoclonal	Abcam	ab8226	1:10,000
CBS	Rabbbit polyclonal	Abcam	ab135626	1:250
CHIP	Rabbit monoclonal	Abcam	ab134064	1:10,000
DHFR	Rabbit monoclonal	Abcam	ab133546	1:1,000
GART	Rabbit polyclonal	In-house	[5]	1:10,000
GNMT	Rabbit polyclonal	In-house	[17]	1:4,000
HSP90	Mouse monoclonal	Abcam	ab13492	1:1,000
MTHFD1	Rabbit polyclonal	In-house	[5]	1:10,000
MTHFR	Rabbit monoclonal	Abcam	ab203786	1:1,000
SHMT1	Rabbit polyclonal	Cell Signaling	12612	1:1,000
TYMS	Rabbit monoclonal	Cell Signaling	9045	1:1,000
Ubiquitin	Rabbit monoclonal	Abcam	ab140601	1:2,500

*For in-house antibodies, corresponding references are from the reference list in the main manuscript.
