# Supplementary material for: CHIP E3 ligase mediates proteasomal degradation of the proliferation regulatory protein ALDH1L1 during the transition of NIH3T3 fibroblasts from G0/G1 to S-phase
Source: PLoS One. 2018 Jul 6;13(7):e0199699. doi: 10.1371/journal.pone.0199699 (PMC6034817; doi:10.1371/journal.pone.0199699)
Supplement: S1 Fig — Samples for the assay were prepared from cells collected at indicated time points (hours) after splitting the culture. Average of three independent experiments and standard errors are shown. This experiment was repeated multiple times (more than ten experiments total) with essentially the same outcome. (PDF) [file pone.0199699.s002.pdf]

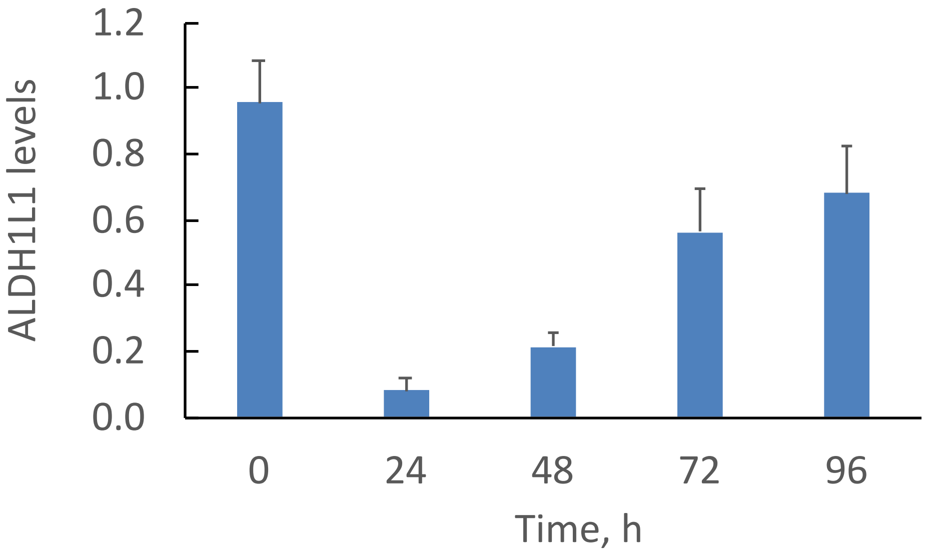

S1 Fig. Relative content of ALDH1L1 protein in NIH3T3 cells from quantification of Western blot bands (normalized to actin; representative image is shown in Fig 1B). Samples for the assay were prepared from cells collected at indicated time points (hours) after splitting the culture. Average of three independent experiments and standard errors are shown. This experiment was repeated multiple times (more than ten experiments total) with essentially the same outcome.
