# Supplementary material for: CHIP E3 ligase mediates proteasomal degradation of the proliferation regulatory protein ALDH1L1 during the transition of NIH3T3 fibroblasts from G0/G1 to S-phase
Source: PLoS One. 2018 Jul 6;13(7):e0199699. doi: 10.1371/journal.pone.0199699 (PMC6034817; doi:10.1371/journal.pone.0199699)
Supplement: S2 Fig — SDS-PAGE (7, 10 or 12% gels were used depending on the target) was followed by Western blot assays with specific antibody. Equal amount of cell lysates (20 μg of total protein) was loaded in each case. Membranes were stripped and re-probed for actin (not shown) to quantify each band relative to actin (S3 Fig). Samples were prepared from NIH3T3 cells collected at different times after splitting (reflects different confluence and the distribution of cells between cell cycle phases, Figs 1 and 8A and 8B). Lane 1, 0 h; lane 2, 24 h; lane 3, 48 h; lane 4, 72 h; lane 5, 96 h (the extra lane in the AICAR blot marked by asterisk corresponds to 120 h). All in-house polyclonal antibodies were verified in previous publications as indicated in Materials and Methods and S1 Table. (PDF) [file pone.0199699.s003.pdf]

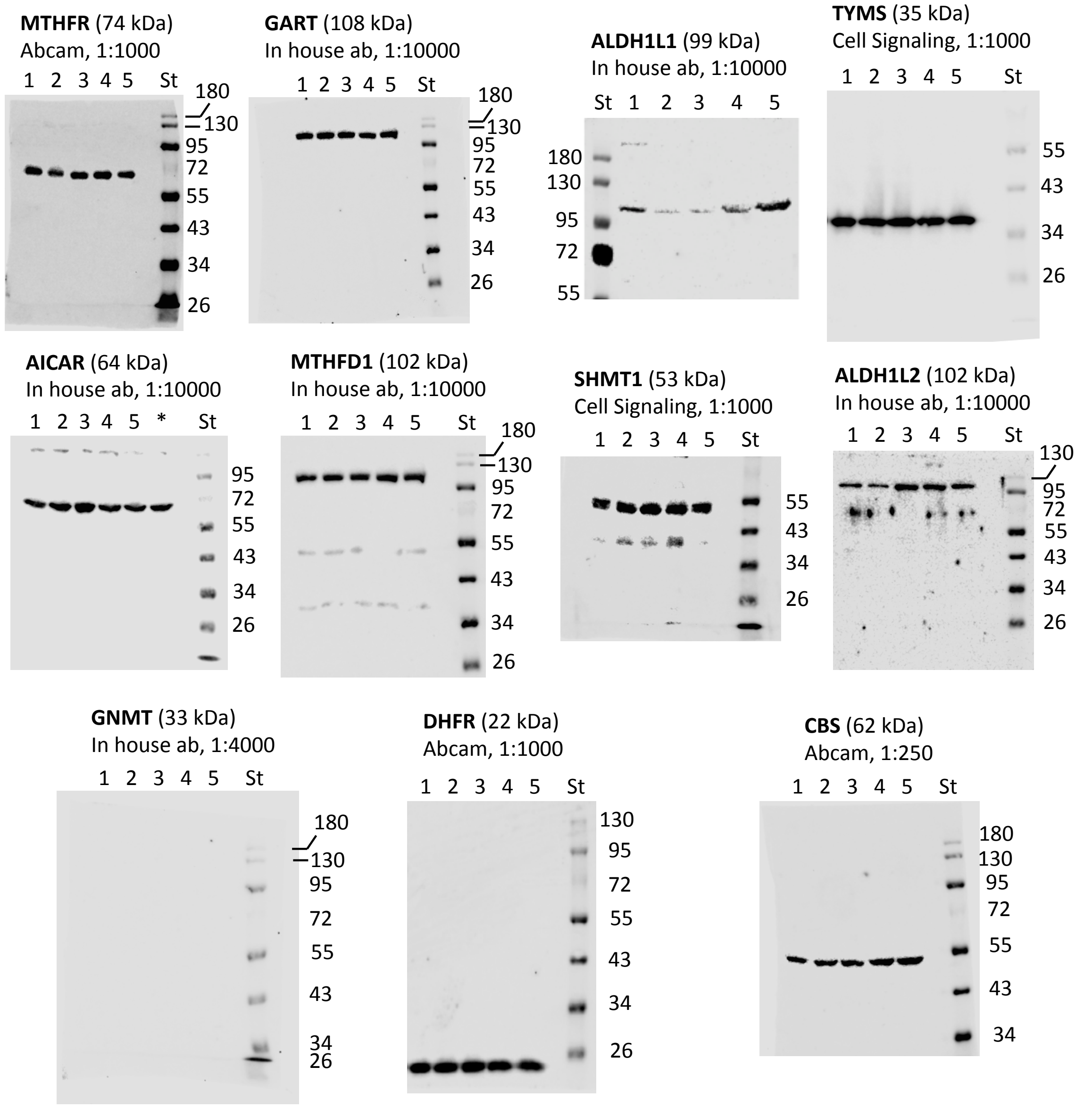

S2 Fig. Western blots of NIH3T3 cells used to create Fig 8D of the main manuscript. SDS-PAGE (7, 10 or 12% gels were used depending on the target) was followed by Western blot assays with specific antibody. Equal amount of cell lysates (20 μg of total protein) was loaded in each case. Membranes were stripped and re-probed for actin (not shown) to quantify each band relative to actin (S3 Fig). Samples were prepared from NIH3T3 cells collected at different times after splitting (reflects different confluence and the distribution of cells between cell cycle phases, Figs 1 and 8A and B). Lane 1, 0 h; lane2, 24 h; lane 3, 48 h; lane 4, 72 h; lane 5, 96 h (the extra lane in the AICAR blot marked by asterisk corresponds to 120 h). All in-house polyclonal antibodies were verified in previous publications as indicated in Materials and Methods and S1 Table.
