# Supplementary material for: CHIP E3 ligase mediates proteasomal degradation of the proliferation regulatory protein ALDH1L1 during the transition of NIH3T3 fibroblasts from G0/G1 to S-phase
Source: PLoS One. 2018 Jul 6;13(7):e0199699. doi: 10.1371/journal.pone.0199699 (PMC6034817; doi:10.1371/journal.pone.0199699)
Supplement: S3 Fig — Samples for the assay were prepared from cells collected at indicated time points (hours) after splitting the culture. (PDF) [file pone.0199699.s004.pdf]

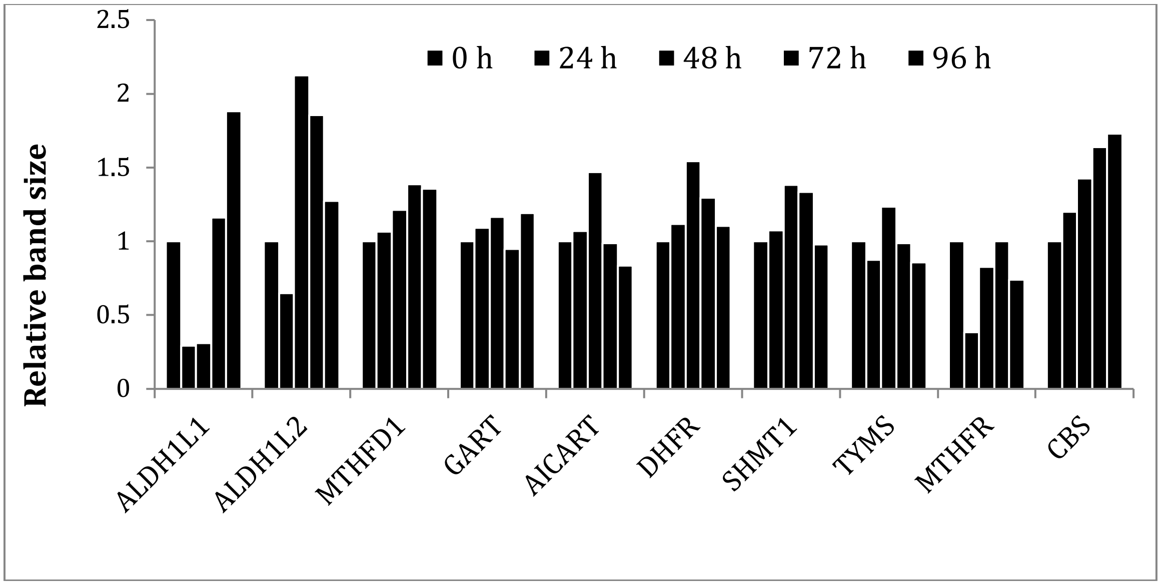

S3 Fig. Plotted values from quantification of bands (normalized to actin) from S2 Fig (also Fig 9D in the main manuscript). Samples for the assay were prepared from cells collected at indicated time points (hours) after splitting the culture.
